# Rehabilitation and Management of Complex Multiple Para-Symphysis Mandible Fracture: A Case Report

**DOI:** 10.7759/cureus.31180

**Published:** 2022-11-07

**Authors:** Vaishnavi A Hatwar, Chaitanya A Kulkarni, Shubhangi Patil

**Affiliations:** 1 Community Health Physiotherapy, Ravi Nair Physiotherapy College, Datta Meghe Institute of Medical Sciences, Wardha, IND

**Keywords:** rehabilitation, internal fixation, fall, facial trauma, para symphysis fracture, mandibular fracture

## Abstract

Fractures of the parasymphysis occur in 15% of all cases of mandibular fracture. The mandible occupies the lowest portion of the face. The prominent bone of the face is severely injured when the lower face is hit by high impacts with upward or obliquely directed force. In this case study, we discussed the alleged trauma case of a 20-year-old man who fell from a height on August 27, 2022, at about 9:00 p.m. near Wardha. His family took him to the hospital in Wardha due to pain that was aggravated during chewing and swelling in his jaw. On investigation, an X-ray was done, and a left parasymphysis and right-side body fracture were noticed. Later, the patient underwent surgery, where open reduction and internal fixation of the left parasymphysis and the right-side body fracture with plating and intermaxillary fixation (IMF) were done. Then he was referred to the physiotherapy department for therapeutic intervention. Rehabilitation was given to reduce pain and swelling, regain full range of motion, gradually increase mobility, and keep associated muscles strong. A six-week protocol was administered to the patient. It was found that the therapeutic approach was quite effective for the patient.

## Introduction

In young males between the ages of 16 and 30, mandibular fractures related to facial trauma are more prevalent. Up to 70% of all fractures of the face involve the mandible. The most frequent fractures are those of the parasymphysis (35-50%), body (21-36%), condyle (20-26%), and angle (15-26%). Coronoid process (1-2%) and ramus (2-4%) are less common forms of fractures [1]. Across the board and in all age categories, boys are more likely than girls to sustain facial fractures. The most common causes of facial fractures in youngsters include road traffic accidents, sports injuries, and falls [2].

Dingman et al. classified mandibular fractures into several categories. According to the anatomical location, it is classified as symphysis, parasymphysis, body region, angle region, ramus region, condylar process, and coronoid process [3]. Distal to the lower canines, vertical lines encircle the symphysis. When they are not in the midline, fractures in this area are known as par symphysis fractures. Mandibular para-symphysis fractures result in step deformity formation and occlusion loss. The segments tend to draw apart, resulting in a gap or step, due to compression forces acting on the inferior border and tension forces acting on the superior border [4]. Treatment options for a mandible fracture can range from non-operative therapy (such as a soft diet) to closed reduction to open reduction with internal fixation, according to the characteristics of the fracture and the surgeon's care decisions [5]. Reconstruction plates provided better treatment outcomes for fractures with comminuted fragments [6]. Intermaxillary fixation (IMF) is a technique that is used for the reduction and stabilization of mandibular fractures [7]. Following mandibular fracture treatment, complications can include numbness, pain while chewing, malocclusion, and poor wound healing. Following surgery, local wound problems can include wound dehiscence, infection at the surgical site, bone malunion or nonunion, and equipment extrusion. Prior research has linked patient characteristics like smoking and delaying medical care with higher complication rates [8]. Complications from treating a mandibular fracture include periodontal problems, face pain, strain in the masticatory muscles, poor chewing function, chin deviation, restricted mouth opening, malocclusion, and many others. Long-term, these complications could affect the patient's level of quality of life [9].

Physical therapy (PT) after facial trauma greatly contributes to improved function of the injured temporomandibular joint (TMJ). PT helps to relieve pain and restore movement to the jaw. Long-term follow-up is advised, and active and passive joint movements to increase the temporomandibular joint range of motion are considered a crucial part of the post-surgical care of the patients [10]. Physical therapy treats temporomandibular disorders (TMDs) to reduce pain, encourage muscle relaxation, reduce muscular hyperactivity, and regain joint and muscle mobility [11]. After the surgery, the patient complained of poor posture, cervical muscular spasms or pain, and reduced upper limb mobility. These are the possible causes and subjects for treatment [12].

## Case presentation

Patient information

This is a case study of a 20-year-old male patient with a dominant right hand. The patient fell on his face while getting up from sitting on the high table at around 9:00 p.m. on August 27, 2022, near Wardha. The patient complained of pain that was aggravated while eating and talking and swelling on his face. The patient's relatives took him to a private dental clinic in Wardha, where he received initial care. On investigation, an X-ray was done, and a fracture at the left parasymphysis with a right-side body was noticed. The patient was then referred to the oral surgery department at Acharya Vinoba Bhave Rural Hospital (AVBRH), Wardha, India. On September 2, he underwent surgery, which included open reduction and internal fixation of the left parasymphysis and right-side body fracture with plating and intermaxillary fixation was done. According to the pain history, the cause of the pain was trauma, which had a sudden onset, a dull aching nature, and was aggravated by movement. The patient's primary complaints were trismus, restricted mouth opening, pain, and edema on the face. The timeline of these events is mentioned (Table 1).

**Table 1:** Timeline of events.

Events	Dates
Complains of pain in the mandible	August 27, 2022
Visited the hospital with complaints of pain in the mandible while eating and talking	August 28, 2022
Diagnosed with traumatic left para-symphysis and right body fracture	August 29, 2022
Underwent surgery for open reduction and internal fixation of left Para symphysis and right body fracture with plating	September 2, 2022
For further management, referred to physiotherapy on	September 9, 2022

Clinical findings 

A physical examination was done following the patient's consent. The patient appeared to be alert, attentive, and well-oriented in terms of time, place, and people during the physical examination. The patient was lying on his back for the examination. The heartbeat was 76 beats per minute, and the respiratory rate was 18 breaths per minute. On evaluation, there was no pallor, icterus, clubbing, cyanosis, or lymphedema. On observational assessment, a bandage was applied to the suture site. On palpation, the face was swollen from trauma, and the deep cervical muscles in the neck were in spasm. According to the musculoskeletal system evaluation, there was reduced muscle strength in both upper extremities. On manual muscle testing, the bilateral upper limb was graded 4 out of 5. The Numerical Pain Rating Scale (NPRS) was used to measure pain. The rating for pain at the suture site was seven out of 10. Trauma also impacted the mouth opening. A maxillofacial examination revealed swelling and tenderness. On the Therabite scale, the mouth opening was 10 mm during the intraoral examination. There were no significant findings on the respiratory and cardiovascular examinations. Patient’s concern: reduced cervical ROM, decreased upper limb strength, difficulty opening the mouth, difficulty talking and eating, ringing in the ears, dizziness, jaw pain, jaw fatigue, headache, swelling, and bruising.

Diagnosis

Investigations like X-ray tests were used to confirm that the patient had a left parasymphysis and a right body fracture.

Assessment

**Figure 1:**
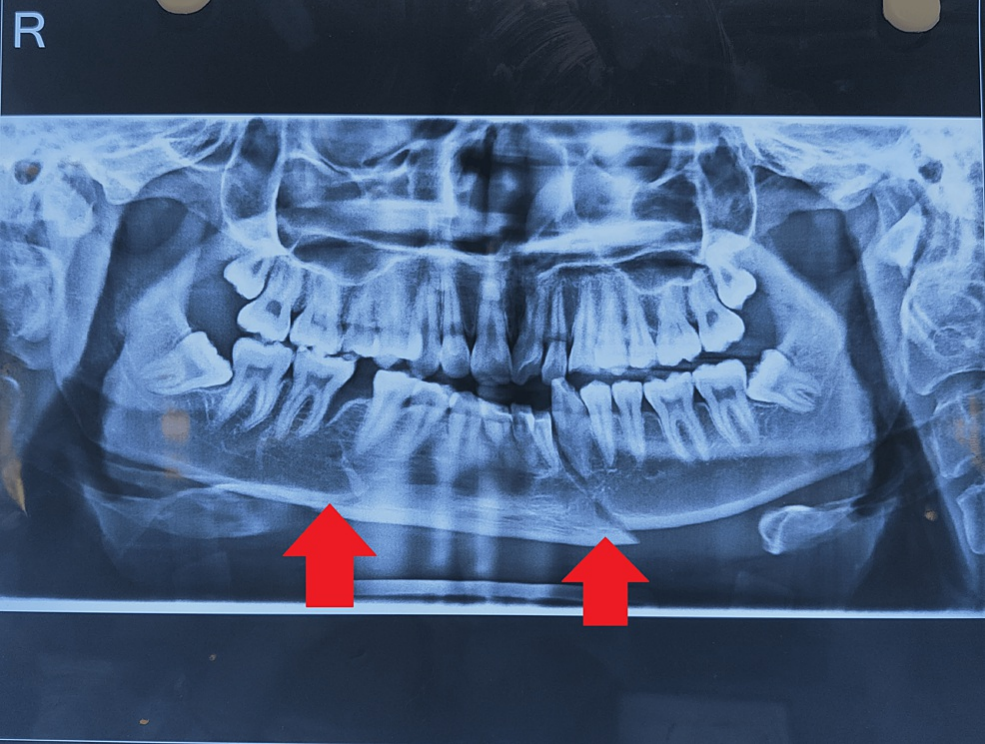
Pre-operative X-ray fracture.

**Figure 2:**
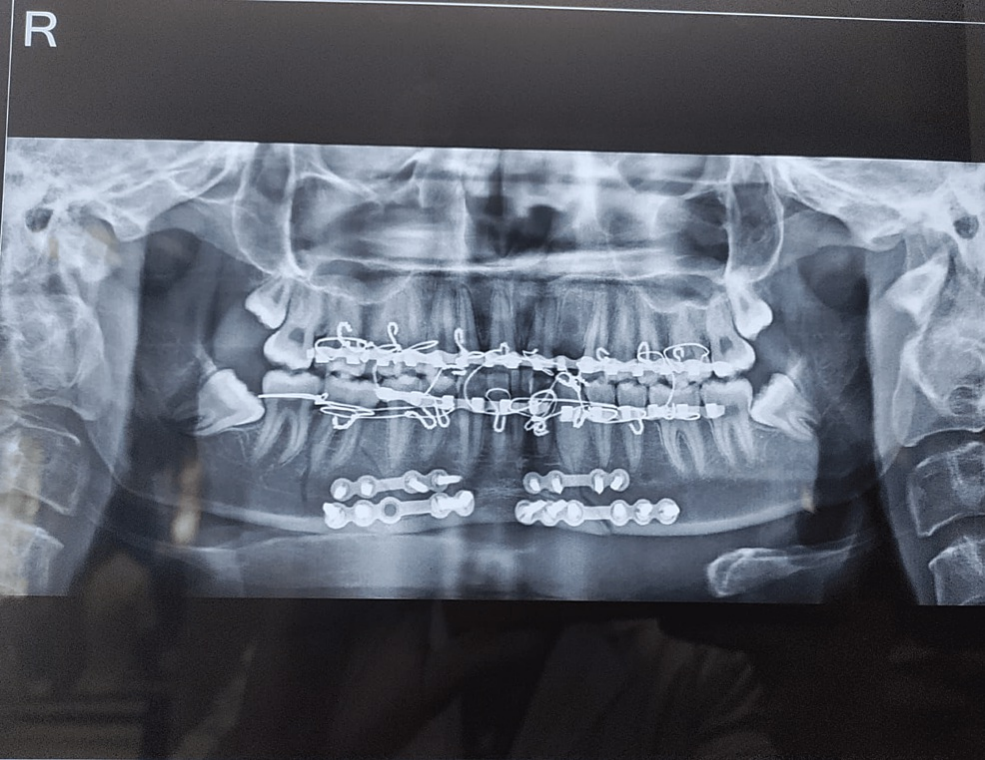
Post-operative X-ray.

Post-physiotherapy assessment: A straightforward and affordable cardboard scale called the TheraBite range of motion scale is intended for patients with TMJ issues to use to test their trismus [13]. TMJ (mouth opening): By using the Therabite scale on the second day of surgery, it was 10 mm (Figures 1-2).

Medical treatment

Primary treatment was done in a private dental hospital at Wardha. On investigation, an X-ray was done, and a fracture was noticed at the left parasymphysis and the right-side body. The patient was then referred to the oral surgery department at AVBRH, Wardha, India. On September 2, he underwent surgery, which included open reduction and internal fixation of the left parasymphysis and right body fracture with plating and intermaxillary fixation. Post-operative medication instructed drug injection (Inj) tramadol 50 mg, Inj Emeset 4 mg intravenous (IV) TD, Inj Pantoprazole 40 mg OD 5 days, antibiotics Augmentin and IV Metro 500 mg, Diclofenac and IV Perinorm, and other supplements: tablet (tab) vitamin C 500 mg BD, tab limcee 500 mg BD, tab Supradyn OD, and tab Chymoral Forte 1 tab TDS.

Therapeutic management

**Figure 3:**
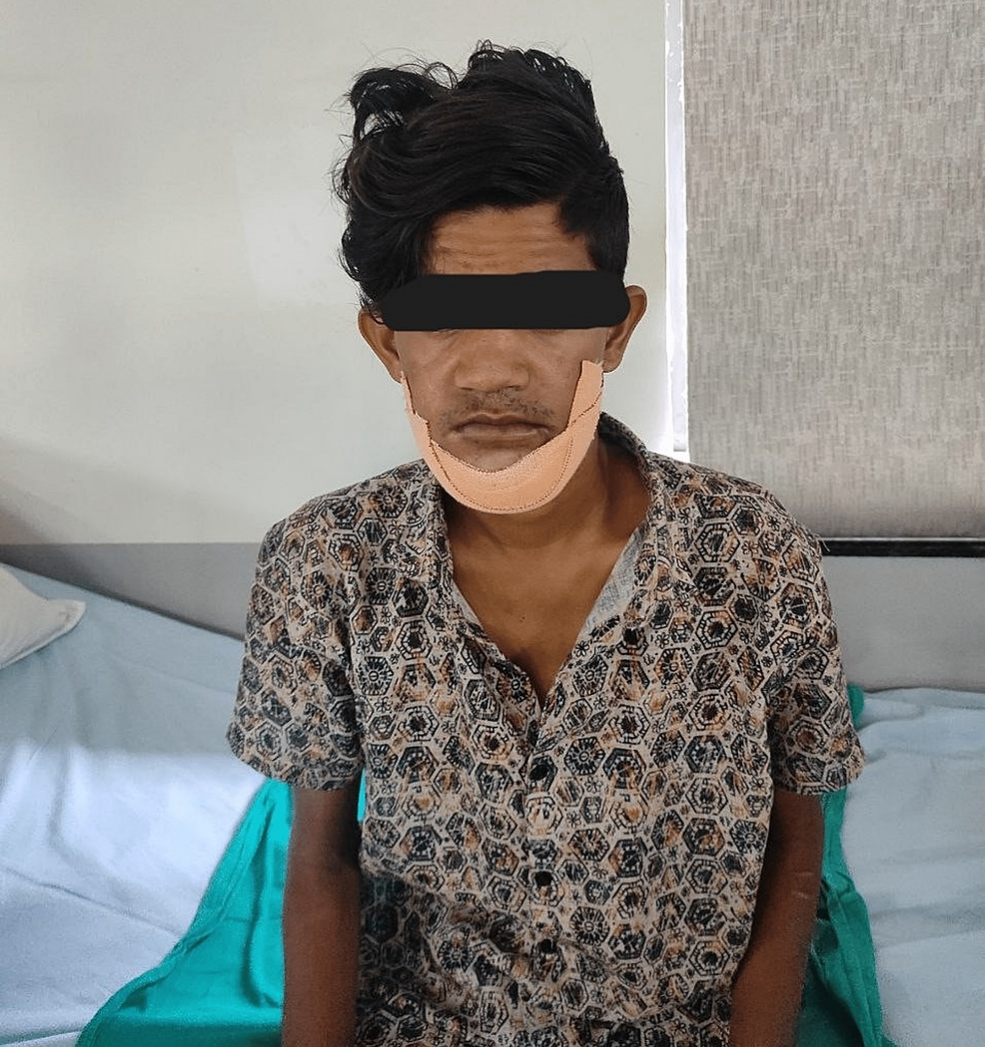
Patient is in sitting on a plinth with a forward head and slouched posture.

**Figure 4:**
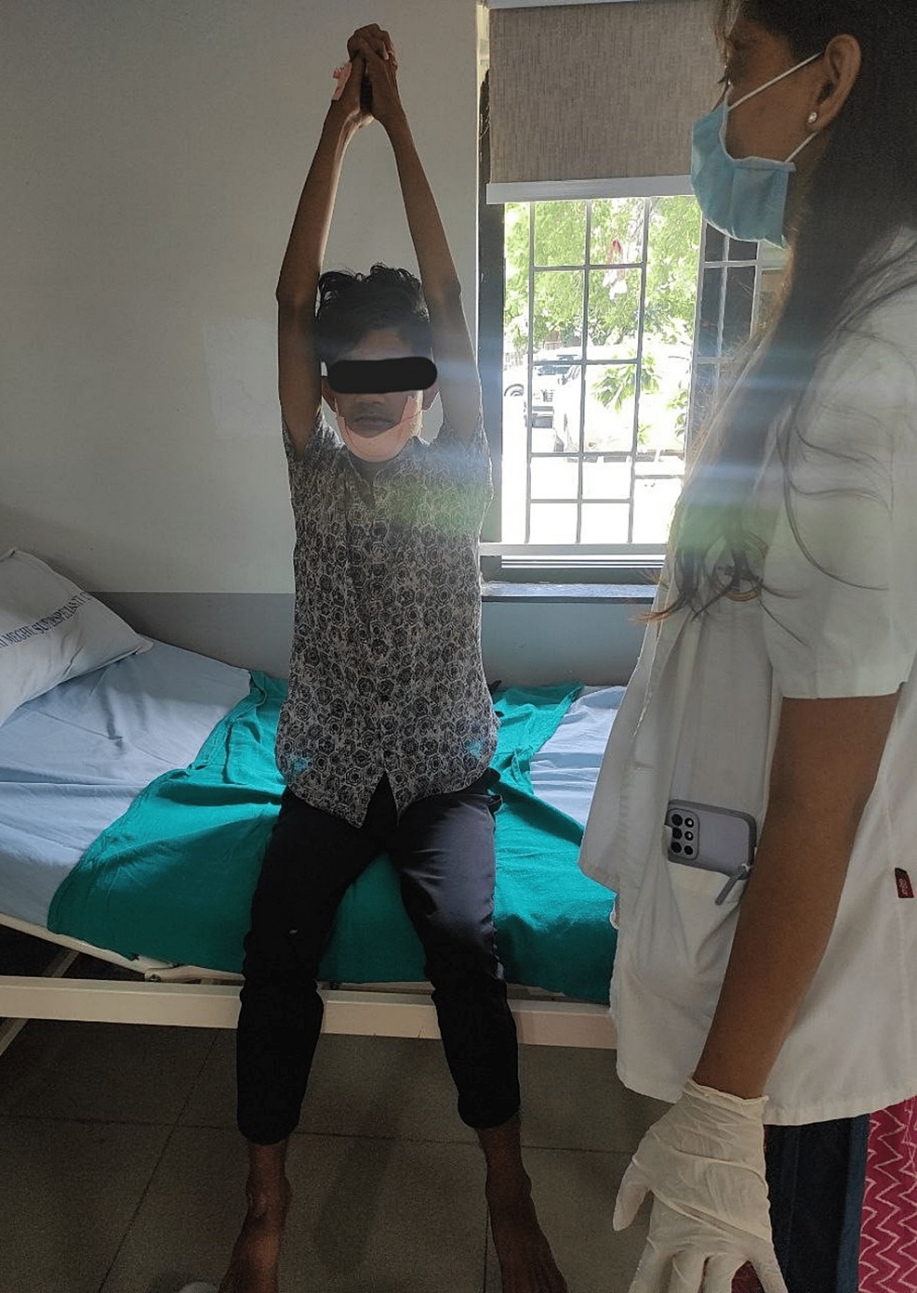
Patient is performing a thoracic expansion exercise.

The physiotherapy management was given for six weeks. The treatment protocol for one to six weeks is explained in Table 2. Figures 3-4 show the posture and exercise performed by the patient.

**Table 2:** The treatment protocol followed for one to six weeks.

Weeks	Goals	Physiotherapy intervention
Week 1 – 2	Patient education. To reduce pain and swelling. To prevent respiratory complications. To enhance mouth opening and decrease trismus. To prevent forward neck posture and slouched posture. To improve the upper limb mobility.	The patient was educated about the condition, instructed on what precautions to be taken, and the role of physiotherapy in this condition, like how physiotherapy is going to help in recovering his state was explained to the patient. Cryotherapy was given to the patient for 10 minutes three times a day following surgery which help to reduce swelling. Breathing exercises such as diaphragmatic breathing, deep breathing exercise, and thoracic expansion exercise 1 set of 10 repetitions (reps) three times a day were given. Mouth opening exercise with ice cream sticks one set of 10 reps given. Mouth opening, blowing, jaw deviation, mouth blowing, and puffing exercises were also given. To improve posture braces, stretching and exercise are given such as door frame stretch exercise, arching over a foam roller, and shoulder blade squeeze. Active range of motion of shoulder joint bilaterally in sitting position.
Week 3–4 (the same protocol as given above and a few additional interventions)	To improve the strength of the facial muscles. To relieve the neck muscles tightness. To improve muscle strength of the upper limb.	Resistance-based oral motor exercises for the jaw were administered. 1 set of 10 reps of each exercise was performed. 3 sessions of myofascial release (MFR) for the upper trapezius muscle were administered on alternate days. Following each session, cryotherapy was given. Isometrics exercise for upper limb muscles were given
Week 5-6 (the same protocol as for weeks one to four and a few additional interventions)	To improve facial muscle strength and to treat trismus.	To improve strength muscle energy technique (MET) was given: one set of five reps was employed. Therapeutic jaw exercises: Two sets of 10 reps were given.

Follow-up and outcome

**Figure 5:**
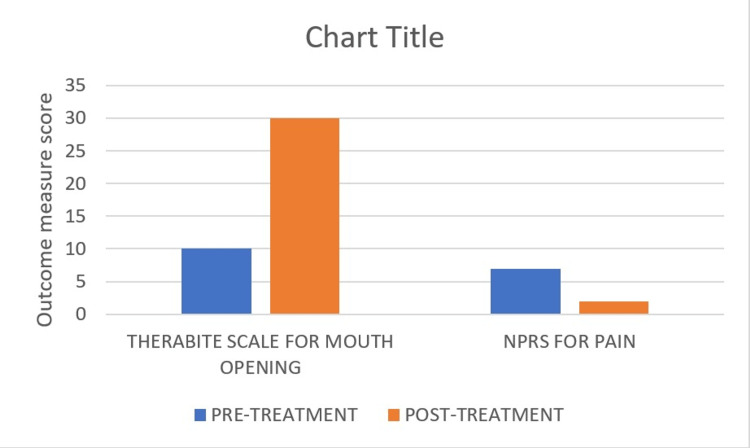
Outcome measure scores of the pre-treatment and post-treatment. Mouth opening distance - Therabite scale method: For measuring the distance, we used a direct approach. The patient was instructed to open their mouth as much as they could. A Therabite scale was placed between the mandibular and maxillary central incisors three times to measure the distance, and the mean of the three measurements was used to determine the measurement's value. The NPRS: It is a unidimensional measure of pain intensity. A ten-point scale is used, with 0 denoting "no pain" and 10 denoting "worse pain.”

After six weeks, the patient came for a follow-up. He had no pain and was able to carry out all daily tasks. The patient was motivated, educated, and strong enough to complete the physical therapy. Additionally, the patient received information on home exercise regimens, and a postural adjustment was suggested. On postoperative day 2, the score was 7/10 on the NPRS. Improvements in NPRS were estimated to be 2/10 following physical therapy. On the Therabite scale, there was a 10 mm mouth opening on postoperative day 2, which progressed to 30 mm on the follow-up assessment day. An increased cervical range of motion in the patient was a sign of improvement. As the patient advanced through the physical therapy or exercise regimen, improvements were progressively seen (Figure 5).

## Discussion

This study provides a platform for expanding the evidence base for physiotherapy intervention for the treatment of maxillofacial conditions. Previous studies investigating the effects of post-mandibular fracture exercise regimens had varying quality levels, dosages, and exercise types. They were also all conducted by surgeons without physiotherapy demonstrations or interventions [14]. A well-organized physical therapy intervention is necessary for treating trismus. A case study by Rasotra demonstrates the significant positive outcome of physiotherapy treatment for a patient with trismus [15].

A study by Armijo-Olivo et al. said that one of the most often used therapies for TMD is physical therapy, which aims to reduce neck and jaw pain while enhancing the range of motion and encourage exercise to preserve healthy function [11]. According to the study by Wang et al., patients demonstrated statistically significant improvements in cervical range of motion, pain relief, physical performance measures, and the degree of disability after an average of four weeks of physical therapy intervention [16]. Patients with acute neck sprains following facial trauma benefited from physical therapy [17]. Active mobilization exercises help in the early healing of fracture sites with good functional ranges in mouth opening [9]. All exercises should be done in front of a mirror. All other physiotherapeutic procedures can be started while the patient is still in the hospital, but jaw muscle stretches and passive TMJ motions should only be started during outpatient follow-up [18].

However, research on physiotherapy intervention in mandibular fractures is lacking. Physical therapy helps increase the functional ability of patients with mandibular fractures. In this study, we discussed a 20-year-old male patient who had an alleged case of trauma due to a fall from a height; he visited the hospital with his family. After an investigation, he was diagnosed with a left parasymphysis and a right body fracture. It was managed operatively through open reduction and internal fixation with plating. After that, a physiotherapy protocol is started from the second day of surgery until the patient continues his previous activities like chewing, eating, talking, and cervical movements. The patient's quality of life was significantly improved by the six-week intervention.

## Conclusions

It was found that the therapeutic approach was quite effective for the patient. The primary complaints of the patient were trismus, limited mouth opening, pain, and swelling on the face. On these problems, we concentrated. We combined various physiotherapy methods to improve and eliminate each issue. As a result of our efforts, we were able to successfully eliminate these concerns, which enhanced the patient's overall quality of life. Physical therapy as an adjunct to surgery was beneficial in preventing post-operative complications and improving patient quality of life. However, not many studies demonstrate the efficacy of physiotherapy regimens in patients with mandibular trauma; we have presented a six-week planned protocol that has been beneficial in such patients. According to the results of our study, the patient with a parasymphysis and right body fracture responded effectively to our therapeutic intervention.
